# Does each Component of Reactive Oxygen Species have a Dual Role in the Tumor Microenvironment?

**DOI:** 10.2174/0929867331666230719142202

**Published:** 2023-08-10

**Authors:** Siyu Hao, Dan Cai, Shuang Gou, Yan Li, Lin Liu, Xiaolong Tang, Yu Chen, Yueshui Zhao, Jing Shen, Xu Wu, Mingxing Li, Meijuan Chen, Xiaobing Li, Yuhong Sun, Li Gu, Wanping Li, Fang Wang, Chi Hin Cho, Zhangang Xiao, Fukuan Du

**Affiliations:** 1 Laboratory of Molecular Pharmacology, Department of Pharmacology, School of Pharmacy, Southwest Medical University, Sichuan Luzhou 646600, China;; 2 Cell Therapy & Cell Drugs of Luzhou Key Laboratory, Sichuan Luzhou, 646000, China;; 3 South Sichuan Institute of Translational Medicine, Sichuan Luzhou 646600, China;; 4 School of Biomedical Sciences, Faculty of Medicine, The Chinese University of Hong Kong, Hong Kong, China;; 5 Department of Oncology, Affiliated Hospital of Southwest Medical University, Sichuan Luzhou 646600, China

**Keywords:** ROS, tumor, microenvironment, molecule, metabolism, therapy

## Abstract

Reactive oxygen species (ROS) are a class of highly reactive oxidizing molecules, including superoxide anion (O_2_^•−^) and hydrogen peroxide (H_2_O_2_), among others. Moderate levels of ROS play a crucial role in regulating cellular signaling and maintaining cellular functions. However, abnormal ROS levels or persistent oxidative stress can lead to changes in the tumor microenvironment (TME) that favor cancer development. This review provides an overview of ROS generation, structure, and properties, as well as their effects on various components of the TME. Contrary to previous studies, our findings reveal a dual effect of ROS on different components of the TME, whereby ROS can either enhance or inhibit certain factors, ultimately leading to the promotion or suppression of the TME. For example, H_2_O_2_ has dual effects on immune cells and non-cellular components within the TME, while O_2_^•−^ has dual effects on T cells and fibroblasts. Furthermore, each component demonstrates distinct mechanisms of action and ranges of influence. In the final section of the article, we summarize the current clinical applications of ROS in cancer treatment and identify certain limitations associated with existing therapeutic approaches. Therefore, this review aims to provide a comprehensive understanding of ROS, highlighting their dual effects on different components of the TME, and exploring the potential clinical applications that may pave the way for future treatment and prevention strategies.

## 1 INTRODUCTION

**Fig. (1):**
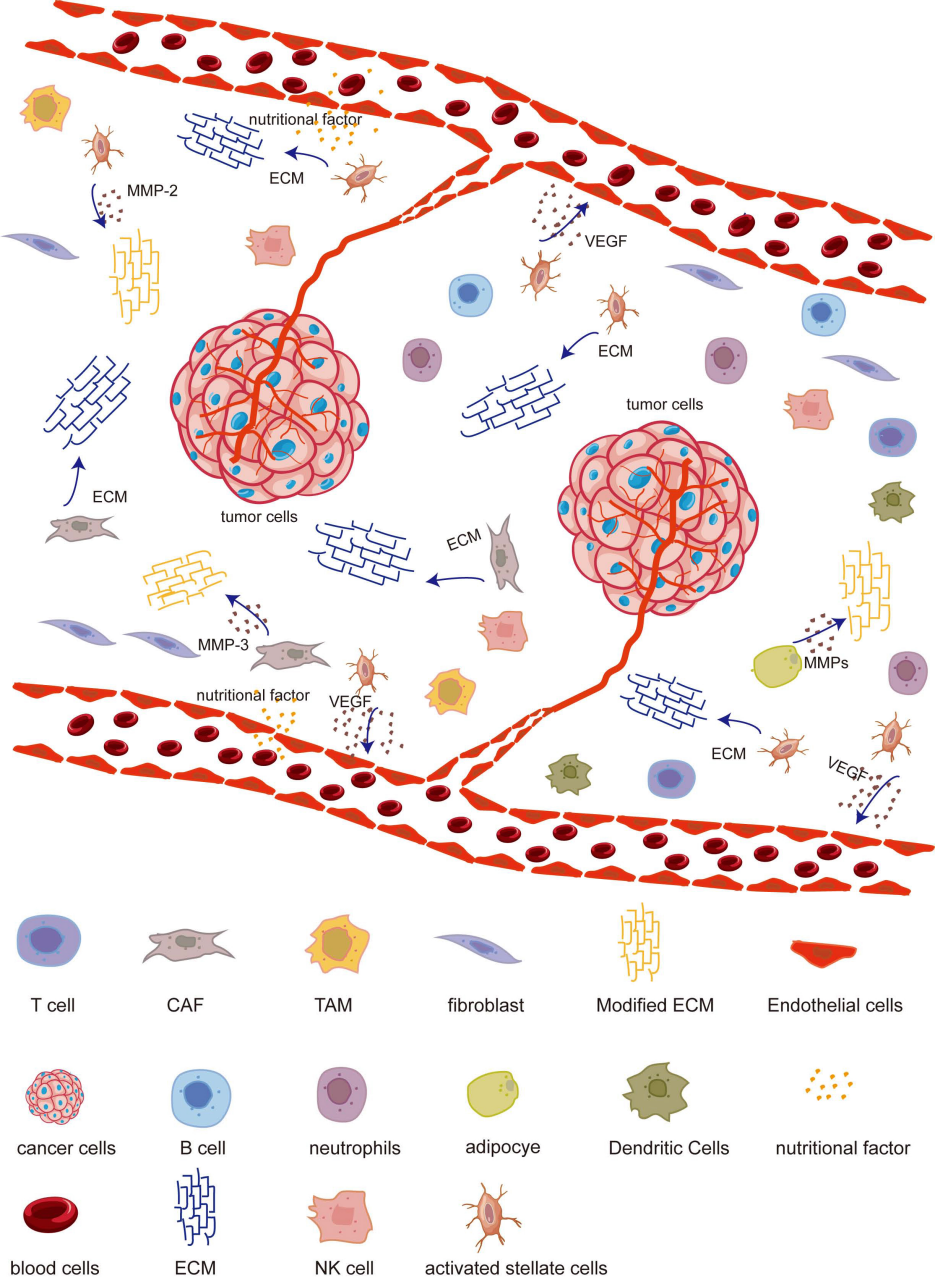
Describes the components of TME and the relationship between various components. TME mainly includes immune cells, stromal cells, and non-cellular components such as ECM. The components of TME coordinate with each other and are closely related.

Tumor cells, immune cells, stromal cells (including vascular endothelial cells, fibroblasts, adipocytes, and stellate cells), and non-cellular components (such as the extracellular matrix (ECM), exosomes, and blood vessels) constitute the major constituents of the tumor microenvironment (TME) (Fig. **1**). However, the specific composition of these elements can vary depending on the tumor type [1, 2]. Furthermore, the tumor microenvironment (TME) and metabolic reprogramming are recognized as significant characteristics of modern cancer research [3]. The interplay between cancer cells and ROS within the tumor microenvironment govern tumor progression at the intersection of these two pivotal biological phenomena [4-6].

ROS, which are molecules with short lifespans and unpaired electrons, are formed through the partial reduction of molecular oxygen. They encompass oxygen-derived compounds comprising highly labile oxygen radicals such as superoxide (O_2_^•−^) and hydroxyl (HO∙) radicals, which can readily transform into more stable, freely diffusible non-radicals like hydrogen peroxide (H_2_O_2_) and hypochlorous acid. Furthermore, ROS encompasses ^1^O_2_, HOO•, and ClO• [7, 8]. ROS play a crucial role as signaling molecules in regulating the interactions among cellular and non-cellular components within the tumor microenvironment [9]. Nevertheless, recent research has primarily focused on exploring the dual role of ROS specifically within tumor cells [10-12]. A comprehensive examination of the impact of ROS on other components of the TME has not been thoroughly conducted. The primary focus of this paper is to investigate the dual effects of ROS on the various components of the TME.

ROS can create a “favorable” environment for tumor cells by modulating the TME, which is characterized by acidity, hypoxia, nutrient deprivation, and inflammation [13-15]. However, the role of ROS in the tumor microenvironment is multifaceted. This review comprehensively examines the generation, characteristics, and functions of ROS, as well as their impact on cancer development. Initially, it provides an overview of the sources of ROS, encompassing the mitochondrial respiratory chain, NADPH oxidases, and other enzymatic systems, while elucidating the properties and structures of ROS. Subsequently, it delves into the intricate interplay between ROS and the TME. Furthermore, it consolidates the current strategies for ROS-targeted cancer therapy and sheds light on their inherent limitations. Lastly, it synthesizes and discusses the patterns and extent of interactions between ROS and the TME, with the ultimate aim of exploring potential clinical applications.

## 2 THE ORIGINS, COMPOSITION, AND CHARACTERISTICS OF ROS, AS WELL AS THEIR PROPERTIES WITHIN THE TUMOR MICROENVIRONMENT

### 2.1 The Origin, Structure, and Properties of H_2_O_2_

The generation of endogenous H_2_O_2_ in cells is intricately associated with mitochondrial activity, the endoplasmic reticulum, and NADPH oxidase [16]. The superoxide radical is produced within the constituents of complex I and complex III of the mitochondrial electron transport chain [17]. Moreover, the superoxide radical can serve as a precursor for the production of H_2_O_2_ [18], which is then converted to H_2_O_2_ through the action of manganese superoxide dismutase (MnSOD) [19]. H_2_O_2_-producing enzymes are also present in the lumen of the endoplasmic reticulum. The most prevalent enzyme source is the flavoprotein ERO1, which collaborates with members of the protein disulfide isomerase (PDI) family of oxidoreductases. ERO1 facilitates the formation of disulfide bonds during the folding of newly synthesized polypeptides. Electrons released during disulfide bond formation are transferred from the nascent secretory protein to ERO1 *via* PDI. Subsequently, H_2_O_2_ is generated as ERO1 transfers the received electrons from PDI to molecular oxygen [20]. NADPH oxidase facilitates the transfer of electrons from NADPH to two distinct heme moieties through flavin adenine dinucleotide. This electron transfer process occurs towards molecular oxygen, serving as the electron acceptor. The resulting multimeric complex ultimately generates either superoxide or H_2_O_2_ [21]. Likewise, superoxide is catalyzed by superoxide dismutase (SOD), leading to the subsequent production of H_2_O_2_ [22]. Therefore, the collective production of H_2_O_2_ in the mitochondria, endoplasmic reticulum, and NADPH oxidase contributes to the overall oxidative stress response in the organism.

H_2_O_2_, with the chemical formula H-O-O-H, possesses a polar molecule structure consisting of both polar and nonpolar bonds. The oxygen atom (O) in H_2_O_2_ carries an intermediate valence of -1. Due to its specific spatial arrangement, H_2_O_2_ adopts a permanent dipole conformation, resulting in the molecule having a distinct separation of positive and negative charges [23]. Furthermore, the presence of a permanent dipole in H_2_O_2_ may have implications for cellular motor function and transport processes [24]. This structural characteristic is intricately linked to the impact of H_2_O_2_ on the invasion and metastasis of cancer cells as described below.

The properties of H_2_O_2_ in the microenvironment encompass its oxidizing, conductive, and transformative characteristics. H_2_O_2_'s inherent oxidative activity is manifested through the degradation of hemoglobin, enzyme inactivation, and release of iron, as well as the oxidation of lipids, DNA, ketones, and -SH groups [25]. When H_2_O_2_ induces oxidative stress in various components of the tumor microenvironment, it can generate a diverse array of “nutrients” that promote tumor growth. H_2_O_2_ demonstrates excellent conductivity within the TME and may be the most suitable oxygen metabolite for signaling among various oxygen metabolites. This may be attributed to H_2_O_2_'s ability to trigger multiple sensors and pathways during the signaling process, as well as regulate transcription factors at various levels. For instance, H_2_O_2_ synthesizes transcription factors by upregulating transcription or increasing mRNA stability and translation or activates individual genes for DNA binding and nuclear transcription by targeting specific chromosomal regions [26, 27]. Given H_2_O_2_'s numerous transduction targets and mechanisms, it frequently operates as a messenger in cell proliferation induction, mechanism activation, apoptosis, and anti-apoptosis within the TME. It is noteworthy that H_2_O_2_, in its role as a signal transmitter, induces cellular apoptosis through a concentration-dependent pathway. In a study exploring H_2_O_2_-induced apoptosis in melanoma cells, it was discovered that low concentrations of H_2_O_2_ initially activate the caspase-dependent apoptotic pathway. Conversely, high concentrations of H_2_O_2_ trigger cell death and cancer cell apoptosis *via* a caspase-independent signaling pathway [28]. H_2_O_2_ is also capable of transformation. In a study, overexpression of rat acyl-coenzyme A oxidase (ACOX) was allowed in the non-tumorigenic rat uroepithelial cell line MYP3, leading to high levels of H_2_O_2_ production in the organism. The resulting study demonstrated that H_2_O_2_ exhibited tumorigenicity in thymus-free nude mice, suggesting a malignant transforming effect of H_2_O_2_ on normal cells [29]. These properties are closely associated with the effects of H_2_O_2_ on the TME discussed later in the text.

### 2.2 The Origin, Structure, and Properties of O_2_^•−^

Superoxide (O_2_^•−^), an intermediate reactive oxygen species, is primarily generated in isolated mitochondria, with complex I (ubiquinone oxidoreductase) and complex III (cytochrome bc1 complex; ubiquinone: cytochrome oxidoreductase) serving as the main sites of its production. Complex I, a multi-component complex consisting of 45 polypeptides, functions as the entry point for electrons derived from nicotinamide adenine dinucleotide (NADH) into the respiratory chain [30, 31]. Complex I generate substantial quantities of O_2_^•−^ through two mechanisms. One of these mechanisms involves the reaction of isolated complex I with fully reduced flavin mononucleotide (FMN), resulting in the production of O_2_^•−^. The extent of FMN reduction depends on the ratio of NADH to nicotinamide adenine dinucleotide (NAD) [32]. The rate of superoxide anion generation through complex I is regulated by the NADH/NAD ratio, which governs the electron transfer process [33]. Another mechanism involves the generation of O_2_^•−^ by complex I during reverse electron transport. In this process, a high membrane potential drives electrons to flow backward from coenzyme Q (CoQH2) to complex I, resulting in the reduction of NAD to NADH at the FMN site and the production of O_2_^•−^ [34]. Ultimately, a large amount of O_2_^•−^ is produced from complex-I [35]. The ubiquinone oxidation center within complex III is responsible for the production of superoxide. The cytochrome bc1 complex, which consists of two homodimers, forms two ubiquinone reaction centers from cytochrome b on each side of the membrane [36]. It forms the ubiquinone oxidation center, also known as center P or Qo site, on the positive side of the membrane, and the ubiquinone reduction center, also known as center N or Qi site, on the negative side of the membrane [37]. Moreover, the generation of the superoxide anion occurs in the ubiquinone oxidation center [38].

O_2_^•−^ comprises a single anion and a single radical [39]. The O-O bond length is 1.33 pm, and there is a σ bond and a 3-electron π bond within the ion. The 17-electron O_2_^•−^ is a separable paramagnetic main group ion and is one of many oxygen reduction reaction intermediate species [40].

The O_2_^•−^ possesses oxidizing and signaling activating properties. Its oxidative nature is attributed to its nucleophilic characteristics. When compounds in the tumor microenvironment are present, capable of donating hydrogen (H), such as tocopherol, ascorbic acid, or other positively charged substances, O_2_^•−^ demonstrates strong nucleophilic reactivity. It selectively targets positively charged centers, initiating chemical reactions [25]. The nucleophilic nature of O_2_^•−^ contributes to the induction of oxidative stress in stromal cells. Additionally, O_2_^•−^ possesses the ability to activate signaling pathways that have profound effects on cellular processes. For example, when exposed to O_2_^•−^ and its intermediate oxidizing metabolites produced by phagocytes, the fibrosarcoma QR-32 cell line demonstrates metastatic behavior, indicating the involvement of O_2_^•−^ in signaling and activation pathways. This can be attributed to the role of O_2_^•−^ as a conductor of activating signals, which triggers the activation of nicotinamide adenine dinucleotide phosphate oxidase, ultimately leading to the transformation of QR-32 fibrosarcoma cells into metastatic tumors [41]. Another illustrative example is the stimulation of proliferation in lung cancer cells, wherein O_2_^•−^ serves as an intracellular messenger within the ROS oncogenic signaling cascade. This highlights the involvement of O_2_^•−^ in facilitating cell proliferation and underscores its significance as a key player in intracellular signaling processes associated with cancer development [42]. In cases of leukemia, O_2_^•−^ functions as a messenger for activation signals, initiating crucial signaling pathways such as the mitogen-activated protein kinase (MAPK) and mammalian target of rapamycin (mTOR) upstream pathways. This activation, in turn, leads to apoptosis in leukemic cells, further emphasizing the pivotal role of O_2_^•−^ in regulating cellular processes and influencing disease outcomes [43].

### 2.3 The Origin, Structure, and Properties of HO∙

Hydroxyl radical (HO∙) is produced within mitochondria through the Fenton and Haber-Weiss chemical reactions. In the Fenton reaction (Equation 1), H_2_O_2_ reacts with Fe^2+^ of low valence to generate HO∙, facilitated by the reduction of iron by O_2_^•−^ in the presence of an iron catalyst (Equation 2). Additionally, ligands that promote redox cycling can enhance the chelation of iron catalysts, further influencing the generation of HO∙ [44].

Fe^2+^(ligand)+H_2_O_2_→Fe^3+^(ligand) + OH^-^+ HO• Eq. (**1**)

Fe^3+^(ligand)+O_2_^•−^→Fe^2+^(ligand) + O_2_ Eq. (**2**)

The combined reaction of Equation 1 and Equation 2 is called the iron-catalyzed Haber-Weiss reaction [45]. It is another way to generate HO∙.

Fe^2+^/Fe^3+^

O_2_^•−^+H_2_O_2_ O_2_+OH^-^+HO•

HO• and its secondary product radicals possess one or more unpaired electrons, rendering them highly chemically reactive. As a result, they can engage in redox reactions with various substances within living organisms [46].

In the microenvironment, the HO∙ exhibits oxidative, conductive, and highly reactive properties. HO∙ is known for its strong oxidizing nature and its rapid reactivity with a wide range of inorganic and organic molecules in cells. These include proteins, amino acids, DNA, lipids, metals, and sugars [25]. These characteristics of HO∙ are closely associated with its ability to modify DNA, induce DNA base lesions, and contribute to oxidative stress in the tumor microenvironment [47-49]. HO∙ exhibits signaling properties, as demonstrated by Sumkhemthong's research group. Specifically, HO∙ functions as a signaling molecule that mediates the autophagic response in lung cancer cells. It confers resistance to the apoptotic effect of the drug cisplatin on cancer cells and promotes cell survival [50]. HO∙, being the most active free radical in biological systems, exhibits high reactivity and instability. Unlike the relatively stable O_2_^•−^, HO∙ is a short-lived species that readily interacts with other molecules. In studies involving apoptosis in HeLa, MW451, and 293 cells, it was observed that HO∙ could directly target telomeres, leading to telomere shortening and induction of apoptosis in cancer cells [51].

### 2.4 The Origin, Structure, and Properties of ^1^O_2_

There are two main mechanisms for the production of ^1^O_2_. The first mechanism involves macrophages (including neutrophils, heart cells, and endothelial cells) accumulating in tissues exposed to oxidative stress or in various disease states. In this process, macrophages generate ^1^O_2_ through a spontaneous disproportionation reaction of O_2_^•−^, with the involvement of H_2_O_2_ and NO•. Additionally, the microenvironment plays a significant role in extracellular ^1^O_2_ production, and research has demonstrated that an acidic cellular environment enhances ^1^O_2_ production by macrophages.

**Fig. (2):**
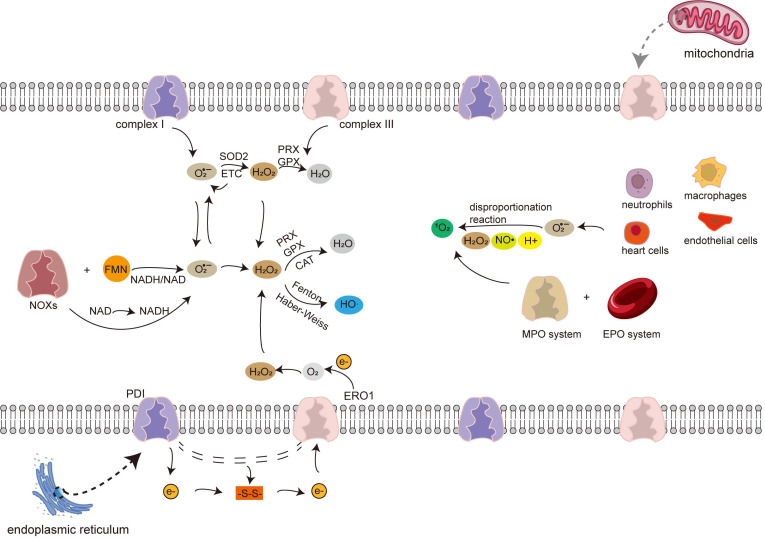
Describes the ROS formation pathway in the body. The superoxide anion is mainly produced in complex I and complex III in mitochondria and relies on NADPH enzymosomes to transfer hydrogen. Hydrogen peroxide is mainly produced by superoxide anion catalyzed by SOD or by electron transfer from ERO1 and PDI enzymes of the endoplasmic reticulum to molecular oxygen. Hydrogen peroxide further undergoes the Fenton and Haber reactions to produce hydroxyl peroxyl. Phagocytic cells, the MPO, and the EPO systems produce singlet oxygen. The four ROS components are not produced independently but have a cascade relationship.

The second mechanism of ^1^O_2_ generation occurs in the erythropoietin (EPO)-H_2_O_2_-bromide system and the Myeloperoxidase (MPO)-H_2_O_2_-chloride system [52] (Fig. **2**).

The excited state of molecular oxygen (^1^O_2_) is characterized by having two paired electrons in its structure and a change in electron spin within the molecular orbital. It is an extremely unstable and highly reactive species [46, 53].

^1^O_2_ is an excited form of molecular oxygen that displays high reactivity towards organic compounds rich in electrons, including polycyclic aromatic compounds, diolefins, and olefins [54]. In a study involving Hepa hepatocellular carcinoma cell lines, it was observed that ^1^O_2_ induced the peroxidation of unsaturated fatty acids within the cells. This lipid peroxidation process has been identified as a direct mediator of iron-induced apoptosis. Consequently, the pathway leading to iron-induced apoptosis in cancer cells can be activated by ^1^O_2_, acting downstream of the cysteine-glutathione axis [55]. Besides, ^1^O_2_ also inactivates SOD and peroxidase on the surface of cancer cells [56, 57], which mediates apoptosis. Due to its exceptional reactivity, ^1^O_2_ exhibits significant cytotoxicity. The oxidation of crucial biological targets, such as lipids, proteins, or nucleic acids, by ^1^O_2,_ can directly trigger cell death [58].

Given the structural and property variations among different ROS components, it is logical to inquire about the potential diversity in their effects on the tumor microenvironment. In the subsequent section, we will thoroughly examine the interplay between each ROS component and the various cellular and non-cellular elements within the TME.

## 3 ROS ALTER THE CELLULAR COMPONENTS IN THE TUMOR MICROENVIRONMENT

### 3.1 ROS Exert an Impact on Immune Cells within the Tumor Microenvironment

#### 3.1.1 ROS Suppress T-cell Function and Facilitate the Establishment of the Tumor Microenvironment

H_2_O_2_ within the tumor microenvironment plays a significant role in promoting the TME by detrimentally affecting T cells, suppressing their immune functions, and impeding their proliferation, thereby facilitating tumorigenic progression. A study by Tan *et al.* illustrated this phenomenon in the context of hepatocarcinogenesis, where Lysyl oxidase-like 4 (LOXL4), secreted by tumor cells and predominantly localized in hepatic macrophages through exocytosis, catalyzes the conversion of amine groups, resulting in the generation of H_2_O_2_ and ammonia as byproducts in a copper-dependent manner. Consequently, H_2_O_2_ activates interferon (IFN)-associated Signal transducers and activators of transcription (STATs), leading to the upregulation of programmed cell death ligand 1 (PD-L1). This process enables macrophages to interact with tumor-killing T cells, hindering T-cell activation, inducing functional impairment, and ultimately suppressing the immune microenvironment [59-61]. In gastric and esophageal cancers, regulatory T (Treg) cells exhibit a notable resistance to apoptosis induced by H_2_O_2_ compared to conventional T cells. This characteristic may result in a higher accumulation of Treg cells in the vicinity of the tumor within the tumor microenvironment, rather than an actual increase in the number of Treg cells influenced by chemical factors [62]. Treg cells possess functional immunosuppressive properties within the tumor microenvironment, effectively suppressing immune responses of T effector cells. Moreover, elevated numbers of Treg cells are observed in various malignancies, indicating their increased presence in the tumor microenvironment [63, 64]. Kono *et al.* conducted a study showing that the secretion of H_2_O_2_ by melanoma-associated macrophages or activated monocytes leads to a decrease in the expression of the cell surface CD3 complex. The CD3 complex plays a crucial role in initiating T cell proliferation and cytolytic function. Based on their findings, the researchers proposed that the inhibitory impact of H_2_O_2_ on T cell function could be attributed to its influence on the expression of the CD3 complex [65]. In an experiment conducted on glioma cells, it was observed that H_2_O_2_ induced the expression of CYB561D2, an antioxidant protein. Additionally, H_2_O_2_ activated signal transducer and activator of transcription 3 (STAT3) in glioma cells. The increased expression of CYB561D2 and activation of STAT3 had immunosuppressive effects on T cells. Specifically, CYB561D2 inhibited the secretion of interleukin-2 (IL-2) from T cells in a STAT3-dependent manner. This immunosuppressive effect of H_2_O_2_ on T cells, mediated by CYB561D2 and STAT3, could ultimately lead to T cell apoptosis and contribute to the immunosuppressive environment in gliomas [66]. IL4I1, a phenylalanine oxidase, can be expressed by infiltrating macrophages and malignant cells in B-cell lymphomas and non-lymphoid tumors [67]. *In vivo*, IL4I1, an immune regulatory enzyme, was found to oxidize phenylalanine, resulting in the production of phenyl pyruvic acid and H_2_O_2_. This H_2_O_2_ generated by IL4I1 exhibited inhibitory effects on T lymphocyte proliferation. Interestingly, H_2_O_2_ had a more pronounced inhibitory effect on memory T cells compared to other T cell subsets. It was observed that H_2_O_2_ at micromolar concentrations had a greater impact on T lymphocyte proliferation inhibition. This suggests that H_2_O_2_, produced by IL4I1-mediated phenylalanine oxidation, plays a role in modulating T cell responses, particularly affecting memory T cells [68]. Indeed, impaired T cell function in tumor-infiltrating lymphocytes (TIL) has been associated with alterations in T-cell receptor (TCR)-mediated signal transduction. In the context of ovarian cancer, Lockhart *et al.* demonstrated that H_2_O_2_ produced by tumor-derived macrophages could contribute to the loss of TCR expression and affect the secretion of high molecular weight factors by certain tumor cells. The loss or decreased expression levels of TCR signaling chains can further impair T cell function, including their ability to recognize and respond to tumor antigens. These modifications in TCR-mediated signal transduction pathways highlight the role of H_2_O_2_ in the tumor microenvironment in influencing T cell functionality within the context of ovarian cancer [69]. Other mechanisms by which H_2_O_2_ interacts with T cells to inhibit TME are listed in Table **1**.

Indeed, O_2_^•−^ plays a role in promoting the tumor microenvironment by inhibiting T-cell proliferation and immune responses. Bronte *et al.* demonstrated that arginase 1 (Arg1) is involved in the mechanism by which myeloid suppressor cells (MSC) inhibit T cell proliferation in colon cancer. Arg1 increases O_2_^•−^ production in myeloid cells through a pathway that may involve the reductase structural domain of inducible NO synthase (iNOS). This increased O_2_^•−^ production is implicated in the Arg1-dependent inhibition of T-cell function. These findings highlight the contribution of O_2_^•−^ and the interplay between myeloid cells and T cells in shaping the immune response within the tumor microenvironment, particularly in colon cancer [70]. Furthermore, additional research has indicated that in cases of melanoma, CD14(+) cells exert a suppressive influence on autologous T cells through a STAT-3, prostaglandin E2 (PGE2), and O_2_^•−^ dependent mechanism. This orchestration of factors not only dampens the immune response but also fosters the progression of tumor development [71].

The impact of ^1^O_2_ on T cells has received limited investigation, with only a singular study demonstrating its capacity to induce mutual oxidative damage to telomeres and mitochondria within T cells. This dual effect of damage is contingent upon both the duration and intensity of exposure, ultimately culminating in T-cell senescence and potentially even fatality [72]. Ultimately, it promotes the tumor cell microenvironment.

#### 3.1.2 ROS Augment T-cell Activity and Impede the Progression of the Tumor Microenvironment

H_2_O_2_ within the tumor microenvironment exerts its influence by augmenting T-cell immunosuppression and cytotoxicity, while concurrently facilitating T-cell activation to repress the tumor microenvironment. In an experimental model employing *in vitro* and *in vivo* approaches, specifically focusing on K-ras-driven pancreatic cancer, Glorieux *et al.* implemented antioxidants to mitigate H_2_O_2_ levels. This intervention significantly attenuated the expression of programmed death-ligand 1 (PD-L1) in K-ras mutant cells, consequently leading to increased T-cell infiltration within tumor tissues. This, in turn, enhanced the immunosuppressive functionality of T cells, resulting in impeded growth of tumor cells [73]. Experimental data indicate that upon exposure of T lymphocytes from mice to mammary cell tumor cells, H_2_O_2_ production occurs. Notably, H_2_O_2_ becomes concentrated in the vicinity of target cells, rendering the sulfhydryl groups on these cells vulnerable to its effects. Through this mechanism, H_2_O_2_ serves as a mediator, amplifying T-cell cytotoxicity and impeding the survival of cancer cells [74]. In a study by Freund *et al.*, it was observed that the treatment of CT26 colon cancer cells with an H_2_O_2_ saline solution resulted in the upregulation of cell surface markers associated with anti-tumor immune response (referred to as ICD). This treatment also promoted T-cell activation. Concurrently, the levels of two anti-inflammatory factors, IL4 and IL10, were found to be elevated. Consequently, the growth of colon cancer cells was inhibited, and apoptosis was induced in the cancer cells [75].

The presence of O_2_^•−^ has been shown to enhance the cytotoxicity of T lymphocytes and suppress the tumor microenvironment. Lu *et al.* employed high-fluence, low-power laser irradiation (HF-LPLI) as a method to induce a significant release of O_2_^•−^. This resulted in the generation of large quantities of O_2_^•−^, which in turn extensively modified the phosphatidylserine fraction of oxidized tumor cells. This modification facilitated macrophage uptake and recognition of the tumor cells, ultimately leading to increased cytotoxicity of T lymphocytes and subsequent suppression of tumor growth [76].

The majority of studies examining the impact of HO∙ on T cells within the TME primarily concentrate on therapeutic applications. These investigations explore therapeutic strategies such as chemodynamic therapy, which involves the utilization of synergetic chemodynamic-photothermo-photocatalytic therapy combined with tetramodal imaging. This approach results in a substantial increase in the production of HO∙ through the stimulation of endogenous H_2_O_2_. Furthermore, HO∙ is implicated in the induction of mitochondrial damage. The reciprocal interaction and cyclic feedback between HO∙ and other ROS significantly augment the presence of cytotoxic cells and helper T lymphocytes in the tumor region. Consequently, these effects contribute to the effective activation of the immune response [77, 78].

#### 3.1.3 ROS Suppress B-cell Function and Foster the Establishment of a Conducive Tumor Microenvironment

Limited research has been conducted on the influence of H_2_O_2_ on B cells within the microenvironment. One notable study by Farber *et al.* provided evidence that in cases of lymphocytic leukemia, B lymphocytes are susceptible to oxidative damage when exposed to H_2_O_2_ in conjunction with T cells. Interestingly, this susceptibility of B cells to oxidative damage was found to be dependent on the presence of Ca^2+^. In contrast, T lymphocytes exhibited relatively higher resistance to the detrimental effects of H_2_O_2_ [79, 80]. The majority of malignant lymphomas (90%) originate from B-lymphocytes within the lymphopoietic system, suggesting a potential link between B-lymphocytes and their increased susceptibility to ROS compared to T cells. However, it is important to note that the existing evidence supporting this hypothesis is limited and lacks substantial strength in the present study. Further research is needed to better understand the differential responses of B and T cells to ROS and their implications in lymphoma development.

#### 3.1.4 ROS Hinder the Activity of NK Cells and Facilitate the Progression of the Tumor Microenvironment

H_2_O_2_ contributes to the promotion of the tumor microenvironment by compromising the activity of natural killer (NK) cells and suppressing immune function. A study conducted by Izawa *et al.* investigated CD56^dim^/CD56^bright^ NK cells in gastric and esophageal cancers and observed that CD56^dim^ NK cells were more susceptible to apoptosis induced by physiological levels of H_2_O_2_ compared to CD56^bright^ NK cells. Furthermore, there was a negative correlation between CD56^dim^ NK cell infiltration in tumors and H_2_O_2_ production. Additionally, H_2_O_2_ was found to impair the antibody-dependent cellular cytotoxicity (ADCC) activity of tumor-associated NK cells [81]. In breast cancer, Klopotowska *et al.* discovered that NK cells exhibit greater susceptibility to H_2_O_2_ compared to T or B cells. As a result, the functionality of NK cells is substantially inhibited, leading to a reduction in their anti-tumor activity. The study also revealed that peroxiredoxin 1 (PRDX1), an essential component of the antioxidant defense system, is deficient in NK cells within the tumor microenvironment. This deficiency in PRDX1 may explain the heightened sensitivity of NK cells to H_2_O_2_ [82]. Furthermore, in melanoma, H_2_O_2_ released by macrophages exerts an inhibitory effect on the promotion of tumor growth by NK cells. It leads to a reduction in the expression of the CD3 complex and CD16 complex in peripheral blood NK cells. This decrease in expression is associated with a decline in the cytolytic activity of NK cells and significantly impairs their effector function [65]. H_2_O_2_ plays a regulatory role in the tumor microenvironment, contributing to tumor growth by inhibiting the function of NK cells and inducing damage to these immune cells.

#### 3.1.5 ROS Enhance the Function of NK Cells and Hinder the Development of the Tumor Microenvironment

H_2_O_2_ can enhance the migration rate of NK cells and exert an inhibitory effect on the tumor microenvironment. For instance, in the context of mast cell tumors, the P815 cell line acts as a “bystander” cell within the tumor microenvironment, releasing H_2_O_2_. This H_2_O_2_ production facilitates the migration of NK cells toward target cells, thereby enhancing the efficiency of NK cells in eliminating these target cells [83]. The impact of H_2_O_2_ on NK cells within the tumor microenvironment is concentration-dependent. Low concentrations of H_2_O_2_ have been shown to enhance the function of NK cells and their effectiveness in suppressing tumors. However, it's important to note that high concentrations of H_2_O_2_ (above 10 μM) can induce apoptosis in NK cells. Therefore, when utilizing H_2_O_2_ to promote tumor suppression through NK cells, careful consideration should be given to maintaining an appropriate and controlled concentration of H_2_O_2_ to avoid detrimental effects on NK cell viability and function.

#### 3.1.6 ROS Regulate the Activity of Macrophages and Promote the Development of the Tumor Microenvironment

H_2_O_2_ plays a role in promoting the tumor microenvironment by impairing the immune function of macrophages and evading their immune response. In the context of hepatocellular carcinogenesis, the expression of LOXL4, an amine oxidase involved in the remodeling of the extracellular matrix, is upregulated. The increased LOXL4 expression leads to the production of H_2_O_2_, which in turn activates STATs associated with interferon (IFN) signaling and induces the expression of programmed cell death ligand 1 (PD-L1). The upregulated PD-L1 expression then acts to suppress the immune function of macrophages, contributing to an immune-suppressive microenvironment in hepatocellular carcinoma [61]. Furthermore, it has been observed that H_2_O_2_ at a concentration of 100μM in lung cancer cells induces oxidative stress, resulting in alterations in the structure of protein subunits. These structural changes may give rise to the formation of novel antigens, which could potentially aid tumor cells in evading the immune response of phagocytes. This phenomenon highlights the ability of H_2_O_2_ to contribute to immune evasion mechanisms employed by tumor cells in the tumor microenvironment [84].

O_2_^•−^, in turn, plays a crucial role in enhancing the expression of C-C motif ligand 2 (CCL2), which serves to recruit macrophages and facilitate the development of the tumor microenvironment. Specifically, in retinoblastoma-deficient tumors, CCL2 expression is upregulated through the activation of AMP-activated protein kinase (AMPK). This activation leads to an increase in fatty acid oxidation, ultimately promoting the production of mitochondrial O_2_^•−^. The activation of the CCL2-CCR2 axis within the tumor microenvironment has been shown to contribute to various tumor types, including sarcoma and breast cancer. This axis serves to facilitate tumor angiogenesis and the recruitment of tumor-associated macrophages (TAMs) as well as myeloid-derived suppressor cells (MDSCs), which are known to exert immunosuppressive effects. Ultimately, these processes regulate the tumor microenvironment, promoting tumor growth in the aforementioned tumor types [85].

#### 3.1.7 ROS Enhance the Function of Macrophages and Inhibit the Development of the Tumor Microenvironment

H_2_O_2_ plays a pivotal role in suppressing the tumor microenvironment by inducing alterations in the phenotype of macrophages. Sang *et al.* employed Iron Nanotrap as a means to target iron transport specifically to tumor-associated macrophages (TAMs) within the tumor. By releasing iron from the Nanotrap, TAMs were reprogrammed and subjected to oxidative stress upon stimulation with H_2_O_2_. This reprogramming process resulted in the conversion of TAMs from a pro-tumor M2 phenotype to an anti-tumor M1 phenotype. Consequently, the reprogrammed TAMs exhibited the ability to elicit an immune response and suppress tumor survival, thereby contributing to the inhibition of tumor progression [86].

Pigment epithelium-derived factor (PEDF) has shown potential in inhibiting the tumor microenvironment by enhancing macrophage activity *via* O_2_^•−^. In the context of prostate cancer, PEDF plays a crucial role in macrophage recruitment and stimulates their polarization towards the classical activation pathway. By regulating the interaction between prostate cancer cells and macrophages, PEDF enhances the immune response of macrophages and promotes apoptosis of tumor cells. Experimental findings suggest that PEDF stimulates the production of O_2_^•−^ by macrophages, which is implicated in the apoptotic process of cancer cells. However, further investigation is necessary to elucidate the precise regulatory mechanism underlying this phenomenon [87].

HO• utilize the disguise of macrophage membranes to confer immune evasion properties to nano-catalysts. Through interaction with the catalyst α4/VCAM-1, macrophages gain the ability to recognize tumor endothelial cells and cancer cells, effectively suppressing the metastasis of breast cancer cells [88].

#### 3.1.8 ROS Enhance Neutrophils and Inhibit the Development of the Tumor Microenvironment

O_2_^•−^ enhances the cytotoxicity of neutrophils against target cells and inhibits the tumor microenvironment. Ishihara *et al.* utilized PSK (a protein-bound polysaccharide) induction to elevate the levels of bronchoalveolar lavage (BAL) neutrophils, resulting in increased production of O_2_^•−^ by neutrophils. This, in turn, led to heightened cytotoxicity of neutrophils towards their targets and a significant reduction in the number and size of metastatic foci in lung cancer cells. However, the precise mechanism through which O_2_^•−^ enables neutrophils to exert cytotoxic effects and inhibit tumor metastasis remains unclear [89]. Furthermore, the specific mechanism by which O_2_^•−^ affects B cells, NK cells, and dendritic cells within the tumor microenvironment (TME) remains uncertain and requires further investigation.

HO∙ exploits neutrophils to facilitate immune responses and suppress the tumor microenvironment. Under conditions where the neutrophil defense system is overwhelmed and the microenvironment provides oxidized iron, neutrophils generate and release HO∙ through iron-catalyzed reactions of superoxide. This process serves to eliminate potential pathogens and mediate the innate immune response [90, 91]. Ultimately, HO∙ inhibits the tumor cell microenvironment.

#### 3.1.9 ROS Exploit Neutrophils to Promote the Development of the Tumor Microenvironment

HO∙ utilizes neutrophils, inducing oxidative stress and ultimately modulating the tumor microenvironment. In cases of gastric cancer associated with H. pylori infection, the presence of virulence factors in H. pylori can attract neutrophils that harbor γ-glutamyl transferase, leading to the generation of different ROS, including H_2_O_2_ and highly reactive HO∙. Prolonged oxidative stress in the infected area results in DNA damage and chronic inflammation, establishing a favorable inflammatory milieu for the survival of tumor cells [92].

### 3.2 ROS Exert a Significant Influence on Stromal Cells Within the Tumor Microenvironment

#### 3.2.1 ROS Enhance the Activity of Endothelial Cells and Benefit the Progression of the Tumor Microenvironment

H_2_O_2_ contributes to the promotion of the tumor microenvironment and sustains tumor cell survival primarily by enhancing the proliferation and migration of endothelial cells. Vascular endothelial growth factor (VEGF) is an important factor involved in angiogenesis, and its upregulation facilitates the migration and proliferation of endothelial cells [93], and endothelial cells provide nutrients to support tumor growth and development [94]. In human prostate cancer, there exists a notable correlation between the expression of VEGF-C, a growth factor with a specific affinity for heparin and vascular endothelial cells, and the occurrence of lymph node metastasis [95, 96]. Muders *et al.* discovered that in prostate cancer, stimulation with H_2_O_2_ leads to an increase in the phosphorylation of serine-threonine kinase AKT-1 and promotes endothelial cell proliferation within cancer cells through the action of VEGF-C. This process renders serine-threonine kinase AKT-1 resistant to inactivation, thereby enabling its continuous activation. The activation of AKT-1 is mediated by mTOR complex 2 (mTORC-2) within the tumor microenvironment, which protects prostate cancer cells from undergoing H_2_O_2_-induced cell death [97]. Furthermore, when exposed to H_2_O_2_, CV-MSCs (mesenchymal stem cells/stromal cells derived from human-term placental chorionic villi) demonstrate a protective effect on endothelial cells, mitigating the harmful impacts of oxidative stress [98]. This phenomenon may be attributed to the modified expression of p53 in CV-MSCs within the breast cancer microenvironment, thereby endowing them with certain capabilities to counteract oxidative stress [99]. In colon cancer, low levels of H_2_O_2_ have been shown to enhance the expression of vascular endothelial growth factors in cancer cells, stimulating the migration and proliferation of endothelial cells. Consequently, this facilitates increased nutrient supply to cancer cells [100].

O_2_^•−^, also known as superoxide, has been found to enhance vascular endothelial cell migration, thereby promoting the tumor microenvironment. David *et al.* employed MALDI-TOF mass spectrometry to identify protein spots associated with melanoma development. Through their analysis, they observed a substantial increase in proteins involved in O_2_^•−^ production, glycolysis, inflammation, and other processes. Furthermore, the induction of O_2_^•−^ resulted in a significant upregulation of VEGF, which further stimulated vascular endothelial cell migration, proliferation, and angiogenesis [101].

#### 3.2.2 ROS Modify the Behavior of Fibroblasts and Reinforce the Progression of the Tumor Microenvironment

H_2_O_2_ plays a role in promoting the tumor microenvironment in fibroblasts by influencing protein expression, oxidative stress, and phenotypic changes. Specifically, in esophageal squamous cell carcinoma (ESCC), H_2_O_2_ induces the upregulation of Hydrogen peroxide-inducible clone 5 (HIC-5) in cancer-associated fibroblasts present in the tumor stroma. HIC-5, in turn, regulates the migration and invasion of ESCC cells by modulating cytokines and altering the ECM. This interplay between H_2_O_2_, HIC-5, fibroblasts, and ESCC cells contributes to the dynamic tumor microenvironment in ESCC [102]. Frequent mutations and deletions of the BRCA1 tumor suppressor gene are observed in hereditary ovarian cancer. In the context of BRCA1-deficient cancer cells, these cells produce elevated levels of H_2_O_2_, which results in oxidative stress and catabolic effects on neighboring stromal fibroblasts. This phenomenon is mediated through the activation of stromal NF-κB, a transcription factor. Consequently, the activated NF-κB pathway leads to the reprogramming of tumor mesenchymal metabolism. This metabolic reprogramming drives mesenchymal-epithelial metabolic coupling, a critical process promoting the development of lethal tumors in the context of BRCA1 deficiency [103]. In human breast cancer, H_2_O_2_ is capable of diffusing easily through the cell membrane and exerting its effects on surrounding fibroblasts. When exposed to acute stress, fibroblasts respond by increasing the expression of Hypoxia-inducible factor-1 (HIF) and chemokine CXCL12. This molecular signaling leads to the transformation of fibroblasts into myofibroblasts, which are characterized by their high contractility and expression of smooth muscle α-actin (SM-α-actin). These activated myofibroblasts play a significant role in promoting the migration and spread of tumor cells within the breast cancer microenvironment [104]. Furthermore, H_2_O_2_ generated by cancer cells elicits oxidative stress in adjacent fibroblasts, culminating in the substantial production of copious quantities of L-lactic acid. This metabolic byproduct serves as a pivotal “fuel” for fostering the proliferation and metabolic activities of breast cancer cells [105]. This intricate process unfolds within the TME of breast cancer, whereby cancer cells emit H_2_O_2_ into the surrounding milieu, instigating oxidative stress and triggering the production of H_2_O_2_ within neighboring cancer-associated fibroblasts (CAFs). Subsequently, these CAFs undergo aerobic glycolysis, generating substantial quantities of energy-dense “fuel” such as ketone bodies, lactate, pyruvate, and fatty acids. Remarkably, this energy-rich “fuel” is then harnessed by cancer cells to sustain their growth and proliferative endeavors [106-108]. Furthermore, the secretion of H_2_O_2_ by CAFs emulates the modus operandi of immune cells such as neutrophils and macrophages, instigating the body's innate immune response mediated by NF-κB. Consequently, this cascade triggers both local and systemic inflammation, thereby fostering an environment conducive to the survival of cancer cells [109]. In the context of prostate cancer, H_2_O_2_ governs distinct translational regulations of matrix metalloproteinase-3 (MMP-3) in both cancer cells and CAFs. Specifically, H_2_O_2_ directly impedes the activity of the MMP-3 promoter in CAFs, consequently suppressing the nuclear translocation of NF-κB and leading to diminished MMP-3 expression in CAFs. Conversely, H_2_O_2_ induces the upregulation of microRNA-128 in prostate cancer cells, which in turn reduces the expression of thrombospondin 2 (a repressor of MMP-3). Consequently, MMP-3 expression in cancer cells is elevated, facilitating the maintenance of ECM homeostasis [110]. Therefore, H_2_O_2_ exerts control over the TME by modulating the behavior of fibroblasts, including cancer-associated fibroblasts (CAFs), thereby shaping the TME to create a favorable environment for tumor growth.

O_2_^•−^ promotes the tumor microenvironment by stimulating autophagy in cancer cells and enhancing the role of fibroblasts. In breast cancer, O_2_^•−^ induces tumor cells to trigger autophagy in neighboring cancer-associated fibroblasts by suppressing Caveolin-1 (Cav-1) expression. Autophagy occurring in the tumor stroma generates high-energy nutrients that support cancer cell metabolism and facilitate their growth [6, 111, 112]. Extracellular superoxide dismutase (EcSOD) serves as the sole extracellular enzyme responsible for scavenging O_2_^•−^. An interesting finding by Golden *et al.* revealed that the overexpression of EcSOD in breast cancer cells had inhibitory effects on hepatocyte growth factor (HGF)-induced phosphorylation of the c-Met receptor tyrosine kinase. This, in turn, resulted in the suppression of cancer-fibroblast interactions and the inhibition of three-dimensional (3D) stromal gel growth of MDA-MB231 cells, a breast cancer cell line. These observations suggest that O_2_^•−^ production enhances HGF-mediated interactions between cancer cells and fibroblasts, thereby promoting the tumor microenvironment [113].

#### 3.2.3 ROS Change the Resilience of Fibroblasts and Deter the Development of the Tumor Microenvironment

H_2_O_2_ plays a suppressive role in the tumor microenvironment by enhancing the stimulation of the Fibroblast-FGF2 system. Fibroblast growth factor 2 (FGF2), a key member of the fibroblast growth factor family, is primarily secreted by fibroblasts. H_2_O_2_, in this context, enhances the activation of the Fibroblast-FGF2 system, thereby exerting suppressive effects on the tumor microenvironment [114]. Ma *et al.* discovered that H_2_O_2_ amplifies the retention of phosphorylated extracellular signal-regulated kinase ½ (pERK1/2) in the cytoplasm and the binding of kinase pGSK3β (Tyr-216) in the peripheral primitive neuroectodermal tumor cell line SK-N-MC, under the stimulation of the Fibroblast-FGF2 system. Furthermore, the experimental findings revealed that the FGF2-induced modulation of phosphorylation and accumulation of pERK1/2 in the cytoplasm exerts a cytotoxic inhibitory effect on SK-N-MC cells, ultimately leading to apoptosis [115]. The impact of H_2_O_2_ on adipocytes and stellate cells within the stromal cells of the TME remains poorly elucidated and necessitates additional research for comprehensive understanding.

O_2_^•−^, through its action on fibroblasts, can upregulate the expression of the tumor suppressor protein p53 and inhibit the fibroblast-mediated proliferation of cancer cells. Wang *et al.* conducted a study on the prostate cancer cell line PC3 and found that fibroblasts secrete basic fibroblast growth factor (bFGF), which promotes the growth of PC3 cells. However, it was observed that a specific concentration of O_2_^•−^ can significantly hinder the proliferation of cancer cells mediated by fibroblast-derived bFGF. This inhibitory effect is attributed to the upregulation of p53 expression induced by O_2_^•−^ [116].

## 4 ROS ALTER THE NON-CELLULAR COMPONENTS OF THE TUMOR MICROENVIRONMENT

### 4.1 ROS Promote Angiogenesis and Enhance the Development of the Tumor Microenvironment

H_2_O_2_, as a signaling molecule produced by the Nox1 gene (a homolog of gp91phox), plays a role in upregulating the protein expression of VEGF and promoting angiogenesis [117]. Nox1 gene expression in tumor cells leads to a significant upregulation of VEGF mRNA expression through the action of H_2_O_2_. This molecular mechanism promotes tumor angiogenesis and enhances the tumorigenicity of DU-145 prostate epithelial cells [118].

O_2_^•−^ plays a role in promoting angiogenesis by upregulating the protein expression of VEGF. Kuroki *et al.* demonstrated that when melanoma and glioblastoma cells were exposed to O_2_^•−^, there was a rapid increase in cellular VEGF mRNA levels. This increase was found to be dose-dependent, meaning that higher levels of O_2_^•−^ resulted in greater VEGF expression. The enhanced angiogenesis induced by O_2_^•−^ contributed to the promotion of tumor growth [119]. Further investigation is required to elucidate the mechanism by which O_2_^•−^, or superoxide, influences non-cellular constituents, including the ECM and exosomes.

HO∙ triggers gene expression and facilitates angiogenesis within the microenvironment. In head and neck squamous cell carcinoma (HNSCC), the oxidative stress induced by HO∙ modifies the expression of BRAK and IL-8 genes in human HNSCC cells *via* the EGFR/MEK/ERK pathway, thereby promoting angiogenesis and facilitating tumor progression [120].

### 4.2 ROS Inhibit Angiogenesis and Impede the Development of the Tumor Microenvironment

H_2_O_2_ functions as an inhibitor of tumor angiogenesis by modifying the protein structure of the VEGF receptor. Peroxiredoxin II (PrxII), an important antioxidant enzyme, plays a crucial role in this process. Dong *et al.* conducted a study revealing that the absence of PrxII resulted in a substantial increase in intracellular H_2_O_2_ levels, leading to oxidative inactivation caused by the altered protein structure of VEGF receptor-2. Consequently, VEGF receptor-2 loses its responsiveness to VEGF stimulation, ultimately inhibiting tumor angiogenesis *in vivo* [121].

### 4.3 ROS Promote ECM Production and Impede the Development of the Tumor Microenvironment

There is limited research exploring the effects of H_2_O_2_ on the ECM. Nevertheless, it has been demonstrated that lysyl oxidase (LOX) plays a role in catalyzing the cross-linking of elastin and collagen within the ECM. This enzymatic process contributes to the regulation of tissue tensile strength [122]. This process plays a crucial role in the migration of cancer cells. Additionally, LOX has been found to facilitate the formation of ECM adhesions and inhibit the migration of invasive breast cancer cells. These effects are mediated by H_2_O_2_ and involve the FAK/Src pathway [123].

The role of ^1^O_2_ in the ECM is not extensively investigated. However, existing studies indicate that ^1^O_2_ interacts with amino acid residues within proteins, generating active substances that facilitate cross-linking of the ECM. This cross-linking process enhances the ECM’s resistance to degradation by matrix metalloproteinases (MMPs), which are responsible for breaking down the ECM and facilitating tumor cell metastasis. As a result, ^1^O_2_ inhibits the migration of invasive tumor cells [124].

### 4.4 ROS Degrade ECM and Facilitate the Development of the Tumor Microenvironment

**Fig. (3):**
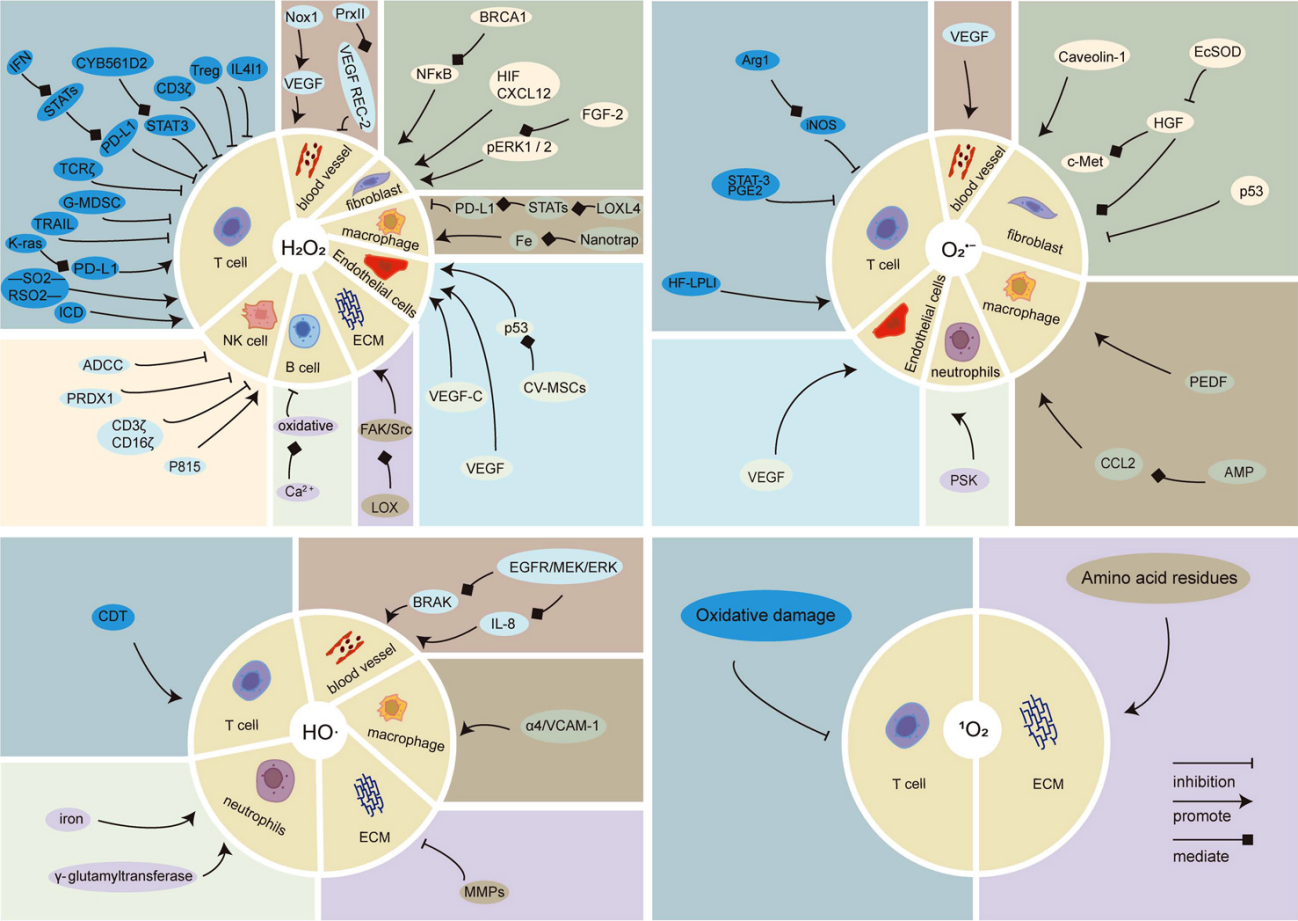
The effect of each component of ROS on the TME component.

HO∙ increases the expression of MMPs, leading to the degradation of the ECM and promoting the tumor microenvironment. Furthermore, HO∙ enhances the process of macromolecular damage to cells mediated by reactive oxygen species and activates MMPs, resulting in heightened invasiveness of cancer cells [125]. However, the investigation of the effects of HO∙ on stromal cells is predominantly lacking in current research (Fig. **3**).

## 5 ANTICANCER THERAPIES INVOLVING ROS MANIPULATION

Based on the previous analysis, it is evident that ROS is a crucial metabolic byproduct within the tumor microenvironment. It participates in tumorigenesis and tumor progression through various mechanisms, including direct cellular damage, promotion of apoptosis, induction of cell proliferation and migration, and modulation of cell signaling pathways. Consequently, research on utilizing ROS for tumor treatment has become a prominent focus in current studies.

Currently, the use of ROS in tumor therapy primarily revolves around several approaches, namely antioxidant therapy, peroxide therapy, targeted ROS therapy, immunotherapy, and combination therapy.

### 5.1 Antioxidant Therapy

Antioxidant therapy is a treatment approach that involves the use of drugs or other methods to reduce excessive free radical activity within the body. Its purpose is to alleviate oxidative stress-induced damage to tissues and organs and to counteract the carcinogenic effects of ROS. This therapy primarily works by inhibiting ROS generation pathways or by enhancing the clearance of ROS through various antioxidants, thus reducing the ROS levels within tumor cells. Carvedilol, for example, exhibits the ability to eliminate ROS generation induced by Benzo(a)pyrene (BaP), thereby safeguarding normal cells from DNA damage and suppressing the malignant proliferation of breast epithelial cells [126]. In line with this, the administration of DHLA (dihydrolipoic acid) effectively prevented the formation of ROS in cells treated with Tetrachlorohydroquinone (TCHQ) and significantly suppressed the development of skin tumors induced by TCHQ [127]. The utilization of antioxidants, either as standalone agents or in conjunction with conventional anticancer medications, holds promise as an effective therapeutic approach to mitigate ROS-mediated tumorigenesis and disease progression in the organism. Extensive data derived from *in vivo* and *in vitro* experiments have substantiated the effectiveness of antioxidants (either alone or in combination) in suppressing tumor cell growth (as summarized in Table **2**).

Nevertheless, certain clinical trials investigating the use of antioxidants for tumor treatment have revealed limitations and, in some cases, even an elevated risk of cancer [128-130]. Several potential explanations can account for this phenomenon. Firstly, the substantial inhibition of oxidative stress can potentially promote the proliferation of cancer cells [131-133]. Secondly, antioxidants have the ability to impede the apoptosis triggered by ROS, which can consequently hinder the normal apoptosis process in cancer cells [134]. Therefore, it is crucial to conduct research aimed at minimizing the potential risk of antioxidants promoting tumor development. This endeavor can lead to the development of more clinically appropriate treatment modalities.

### 5.2 Oxidative Therapy

Oxidative therapy involves further increasing ROS levels to a toxic level, activating multiple cell death pathways, enhancing chemosensitivity, and opening up new avenues for cancer treatment [10]. The primary objective is to elevate ROS levels by utilizing ROS generation inducers and suppressing antioxidants. As an illustration, upregulating p53 expression and downregulating superoxide dismutase 2 (SOD2) expression through the use of Astragalus purpureus led to heightened ROS levels and activation of the mitochondrial apoptotic pathway, effectively impeding the growth and proliferation of liver tumor cells [135]. The efficacy of Deoxyelephantopin in reducing the viability of osteosarcoma cells is dependent on the dosage. It achieves this by promoting the generation of ROS, activating apoptosis-related proteins like Bax, and inducing mitochondrial dysfunction [136]. Several studies and clinical trials have investigated the inhibition of tumor cells by increasing ROS levels (Table **3**). However, it is important to note that solely increasing ROS concentration as a treatment approach for tumors may have undesirable effects. Inducing ROS through certain agents can disrupt the redox balance in tumor cells, potentially leading to DNA damage, cell genome mutations, tumor initiation, or increased survival of tumor cells [137, 138]. Overall, it appears that tumor cells have higher levels of ROS compared to normal cells, which makes anti-ROS therapy a targeted approach for treating tumors. However, achieving the right balance of anti-ROS agents and inducing ROS production in clinical research to achieve therapeutic goals without causing harmful or even contradictory side effects still requires further improvement.

### 5.3 Targeted ROS Therapy

Targeted ROS therapy primarily involves the use of specific targeted molecules such as nanomaterials and small molecules to selectively target specific ROS molecules for treatment. Based on their action sites and mechanisms, targeted ROS molecules can be classified into those targeting ROS generation, ROS signaling pathways, and ROS scavenging, among others. For example, researchers have developed targeted peptide precursor nanodrugs that conjugate with the ROS-sensitive sulfone group, linked with the cytotoxin epothilone B. These nanodrugs exhibit high-level targeting of ROS generation and release epothilone B upon cleavage, demonstrating remarkable tumor selectivity and excellent anti-cancer efficiency in both *in vitro* and *in vivo* studies [139]. Targeted ROS therapy also includes photodynamic therapy and combination chemotherapy [140, 141]. Targeted ROS therapy offers the advantages of strong specificity, significant efficacy, and minimal side effects. By precisely targeting ROS for treatment, this approach can minimize damage to normal cells and improve therapeutic outcomes. Currently, targeted ROS therapy has been widely applied in clinical tumor treatment, particularly for lung cancer, liver cancer, and other types of tumors [142, 143].

Although targeted ROS therapy holds certain application value, it also presents several challenges and limitations. For instance, in certain specific tumors such as melanoma, the presence of melanin enhances oxidative stress defense and may have defective apoptotic pathways, posing a challenge in developing efficient and specific targeted ROS drugs [144]. In summary, targeted ROS therapy holds promise in tumor treatment, but it also faces challenges and limitations. Therefore, future research should focus on exploring highly efficient and specific targeted ROS drugs, as well as investigating their combination with other treatment modalities to enhance therapeutic efficacy and minimize side effects.

### 5.4 Immunotherapy

Immunotherapy utilizes ROS as a target for treatment, aiming to enhance the body's antioxidant capacity and strengthen the immune system's ability to eliminate tumor cells, thereby achieving the goal of tumor therapy. ROS-based immunotherapy encompasses various approaches, including autophagic cell death and inflammation modulators. For instance, a chemical photothermal nano-platform has been developed to activate immune sites using ROS, mediating cancer cell apoptosis and autophagic death to enhance treatment efficacy [145].

Targeting ROS as a therapeutic target in immunotherapy holds significant importance as it can enhance the body's antioxidant capacity and augment the immune system's cytotoxicity against tumor cells, thereby facilitating tumor treatment. However, further understanding of the specific mechanisms and potential risks of ROS in immunotherapy is necessary to provide scientific guidance for its clinical application.

### 5.5 Combination Therapy

Combination therapy refers to the use of two or more different treatment modalities together to enhance therapeutic efficacy and reduce side effects. In the context of utilizing ROS as a target for immunotherapy, combining ROS inhibitors with chemotherapy drugs, immunotherapy agents, or other treatment modalities can enhance treatment effectiveness while minimizing adverse effects [146, 147]. Combination therapies can include ROS inhibitors in conjunction with chemotherapy drugs, ROS inhibitors combined with immunotherapy agents, ROS-releasing agents combined with chemotherapy drugs, and ROS-releasing agents combined with immunotherapy agents.

Although combination therapy can improve treatment outcomes and reduce side effects, it is crucial to consider factors such as drug interactions, dosages, and administration methods. It is important to undertake combination therapy under the guidance of a healthcare professional to ensure treatment safety and efficacy.

## CONCLUSION

Based on the previous discussion, it is evident that many current therapeutic strategies are based on the direct impact of ROS on tumor cells. Moreover, whether it is antioxidation, peroxidation, targeted ROS therapy, or immunotherapy, *etc.*, all exhibit certain drawbacks and side effects. Hence, it is apparent that solely adjusting the ROS levels may not always lead to optimal treatment outcomes. Can we, then, derive novel therapeutic approaches from the relationship between ROS and TME? Firstly, by examining their interactions, we can identify certain patterns and potentially establish a foundation for innovative treatment strategies.

First and foremost, among the various components of ROS, H_2_O_2_ exhibits the most extensive influence on the tumor microenvironment. This can be attributed to the dual impact of H_2_O_2_ on immune cells such as T cells, B cells, NK cells, and phagocytes, as well as stromal cells including endothelial cells and fibroblasts. Additionally, H_2_O_2_ affects non-cellular components such as blood vessels and ECM. In terms of magnitude, the remaining components, namely O^2•−^, HO∙, and ^1^O_2_, also exert dual effects, albeit to a lesser extent. This observation may be attributed to H_2_O_2_'s ability to activate multiple sensors and pathways involved in signal transduction, as discussed in the previous article. For instance, H_2_O_2_ enhances signaling by promoting the upregulation of transcription or by increasing mRNA stability and translation to facilitate the synthesis of transcription factors [26, 27]. Furthermore, due to its ability to target various transduction pathways and mechanisms, H_2_O_2_ offers a unique opportunity to regulate genes and signaling with remarkable precision.

Secondly, H_2_O_2_ displayed the highest sensitivity to the influence of the tumor microenvironment. This is evident from the findings presented in Table **4**, where Zhu *et al.* experimented to investigate the upregulation of H_2_O_2_ on vascular endothelial growth factor. Notably, even at a concentration as low as 10^-3^μM, H_2_O_2_ induced the migration of endothelial cells within the microenvironment of colon cancer [100]. Nevertheless, it should be noted that the other ROS exhibited minimum effective concentrations greater than 10^-3^μM.

Thirdly, it is worth noting that the action concentration of ^1^O_2_ is relatively low. Unlike the other ROS concentrations in Table **4**, which were mostly above 1 μM, there was only one instance of 2x10^-1^ μM MG2I (a chemical fluorogen) used to induce ^1^O_2_ production. From this, we can infer that ^1^O_2_ exerts its effects on the TME under low-concentration conditions. This can be attributed to the low concentration of the inducer MG2I and the volatile nature of ^1^O_2_ itself. Despite its unstable structure and short half-life, ^1^O_2_, being a diolefin with high reactivity, remains effective even at low concentrations [54].

Fourthly, H_2_O_2_ exhibits its effects across a wide range of concentrations. As observed in Table **4**, the action concentrations of H_2_O_2_ in the TME span from 10^-3^μM to 5x10^4^ µM. It is crucial to emphasize that H_2_O_2_ can elicit contrasting effects on the same component of the TME at the same concentration. For instance, different experiments conducted by Bang and Freund demonstrated that 100 µM of H_2_O_2_ yielded opposing effects on T cells, namely enhancement, and inhibition, respectively [66, 75]. However, it is important to consider that the microenvironments of T cells in the two studies were different: one was in the glioma microenvironment, while the other was in the colon cancer microenvironment. Consequently, the same concentration of H_2_O_2_ may yield opposite effects on T cells in different tumor types. Therefore, when conducting research or clinical treatment involving H_2_O_2_, careful consideration should be given to the specific tumor type under investigation.

Fifth, it is noteworthy that the four ROS components do not exhibit dual actions simultaneously on the same component within the TME. For instance, H_2_O_2_ promotes ECM, O_2_^•−^ promotes endothelial cells, HO∙ inhibits ECM, and ^1^O_2_ inhibits T cells. As a result, whether the ROS components have a dual effect on the overall TME environment requires individual investigation and cannot be generalized.

Based on the legislation and the effects of ROS on various components of the TME outlined in Table **1**, it may be possible to select specific ROS treatments based on different tumor types, tumor microenvironments, and concentrations employed. For instance, taking into consideration the abundance of cancer-associated fibroblasts (CAFs) in breast cancer, Agnieszka's research team discovered that H_2_O_2_ excessively activated the JNK1 stress signaling pathway within the cancer stroma. This resulted in the acquisition of a CAF-like phenotype and promoted tumor invasion in breast fibroblasts. Conversely, inhibiting the activation of JNK1 signaling by reducing H_2_O_2_ levels using the peroxidase PRDX1 effectively mitigated breast fibroblast invasion and tumor development [148]. Moreover, the synergistic administration of lactoferrin and black tea polyphenols has been shown to effectively suppress the generation of HO∙ and O_2_^•−^ in hamster buccal pouch carcinoma. This combined treatment strategy exhibits inhibitory effects on tumor angiogenesis, providing a promising approach for impeding the formation of new blood vessels in the tumor microenvironment [149]. Consequently, the treatment of tumors with ROS is not solely centered around directly suppressing malignant cells but also revolves around the pivotal influence of ROS on the protective barrier of tumors, known as the tumor microenvironment.

In pursuing ROS-based interventions for the TME, adherence to three overarching guidelines is warranted. Firstly, drug administration ought to align with the specific tumor type and its distinct microenvironment. Secondly, diverse ROS components manifest distinct impacts, thus cautioning against a generalized approach to ROS dosing. Thirdly, even when dealing with the same ROS, disparate concentrations yield inconsistent outcomes, necessitating vigilance regarding alterations in blood levels.

**Fig. (4):**
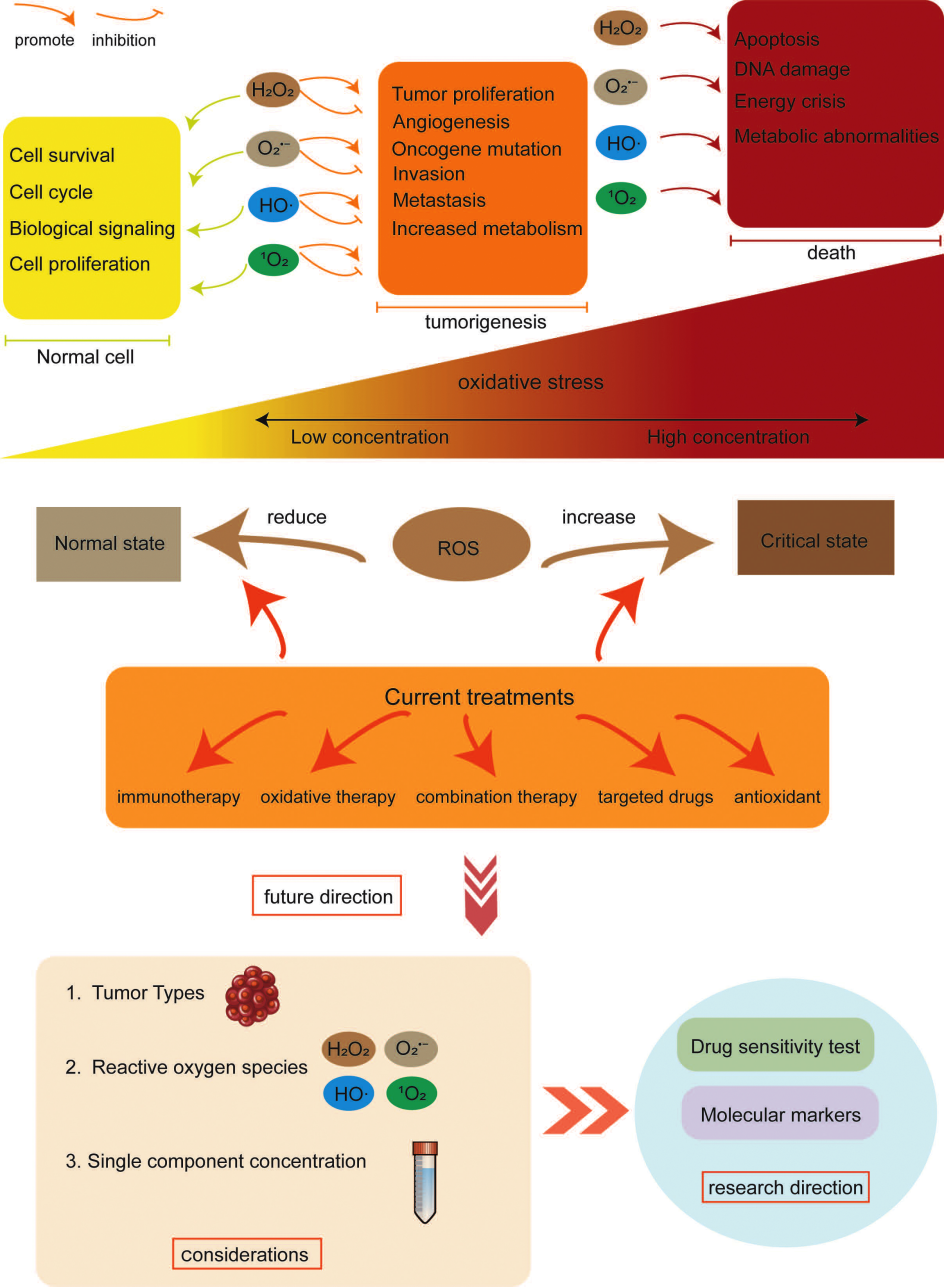
Depicts the change of ROS effect on TME with the change of ROS level. At low concentrations, ROS mainly plays a role in signal transduction. With the occurrence of the tumor, ROS promotes tumor invasion and metastasis, proliferation, and mitochondrial damage. When the concentration of ROS reached a certain level, the apoptosis and DNA damage of cells in the TME environment were induced more. The graph shows existing treatment strategies for this condition. In addition, future research directions are proposed based on the relationship between ROS and TME.

To adhere to these recommendations, several strategies can be employed in the development and utilization of clinical drugs. Primarily, it is advisable to focus on designing drugs that specifically target individual ROS components. Additionally, the utilization of *in vitro* cell/organ drug sensitivity assays can aid in determining the optimal efficacy and dosage concentrations before drug administration. Furthermore, each ROS component should be thoroughly investigated to identify specific dosing markers that provide precise dosing instructions and therapeutic targets (Fig **4**). However, it is worth noting that there is a dearth of therapeutic studies exploring the modulation of ROS in the tumor microenvironment for tumor eradication, despite its promising potential in targeting TME's crucial role in tumor development. Therefore, further research focusing on ROS components with high specificity for the TME is eagerly anticipated to advance effective tumor treatments.

## Figures and Tables

**Table 1:** The effect of each component of ROS on TME and experimental results.

**ROS Type**	**Effect**	**Cancer Type**	**Components**	**Experimental Result**	**References**
H_2_O_2_	promotion	liver cancer	T cells	Impaired immune activation	[59-61]
		gastric and esophageal cancers	Treg cells T effector cells	Suppression of immune response	[62-64]
		melanoma	T cells	Inhibition of proliferation	[65]
		glioma	T cells	Immunosuppression and even apoptosis	[66]
		B-cell lymphomas and non-lymphoid tumors	T cells	Inhibition of proliferation	[68]
		ovarian cancer	T cells	Impaired cellular function	[69]
		squamous cell carcinoma of the head and neck	T cells	Inhibition of proliferation and survival	[150]
		melanoma	T cells	Inhibition of cellular responses	[151]
		colon cancer	T cells	Inhibition of cellular responses	[152]
		astrocytoma	T cells	Apoptosis	[153]
		breast cancer	CD8 T-cells	Inhibition of cytotoxicity	[154]
	inhibition	pancreatic cancer	T cells	Enhancement of immunosuppressive function	[73]
		mammary tumor	T cells	Enhancement of toxicity	[74]
		colon cancer	T cells	Activation	[75]
	promotion	lymphocytic leukemia	B lymphocytes	Oxidative damage	[79, 80]
		gastric and esophageal cancers	CD56^dim^NK cells	Apoptosis	[81]
		breast cancer	NK cells	Reduced activity	[82]
		melanoma	NK cells	Reduced activity	[65]
	inhibition	mast cell tumor	NK cells	Accelerated Migration	[83]
	promotion	liver cancer	Macrophages	Suppression of immune function	[61]
		lung cancer	Macrophages	Dodging the immune response	[84]
	inhibition	/	Macrophages	Immune Enhancement	[86]
	promotion	prostate cancer	Endothelial cells	Promote proliferation	[97]
		colon cancer	Endothelial cells	Promote proliferation	[100]
		esophageal squamous cell carcinoma	Fibroblasts	Induced expression	[102]
		hereditary ovarian cancer	Fibroblasts	Induction of oxidative stress	[103]
		breast cancer	Fibroblasts	Induction of oxidative stress	[104]
		prostate cancer	Fibroblasts	Reduced expression	[110]
	inhibition	nerve tumor	Fibroblasts	Enhanced stimulation	[115]
	promotion	prostate cancer	Blood vessel	Increased angiogenesis	[118]
	inhibition	/	Blood vessel	Inhibition of angiogenesis	[121]
	promotion	breast cancer	ECM	Promote the formation of ECM	[123]
O_2_^•−^	promotion	colon cancer	T cells	Inhibition function	[70]
		melanoma	T cells	Inhibition function	[71]
	inhibition	/	T cells	Increased cytotoxicity	[76]
	promotion	Retinoblastoma sarcoma breast cancer	Macrophages	Regulation of microenvironment	[85]
	inhibition	prostate cancer	Macrophages	Apoptosis	[87]
		lung cancer	Neutrophils	Increased target cytotoxicity	[89]
	promotion	melanoma	Endothelial cells	Promote proliferation	[101]
		breast cancer	Fibroblasts	Promote metabolism	[6, 111, 112]
		breast cancer	Fibroblasts	Facilitated interaction	[113]
	inhibition	prostate cancer	Fibroblasts	Inhibition of proliferation	[116]
	promotion	melanoma glioblastoma	Blood vessel	Angiogenesis	[119]
HO∙	inhibition	/	T cells	Activation of immunity	[77, 78]
		breast cancer	Macrophages	Suppression of metastasis	[88]
	promotion	stomach cancer	Neutrophils	Inflammatory environment	[92]
	inhibition	/	Neutrophils	Promotion of immunity	[90, 91]
	promotion	Squamous cell carcinoma of the head and neck	Blood vessel	Angiogenesis	[120]
		/	ECM	Invasion	[125]
^1^O_2_	promotion	/	T cells	Apoptosis	[72]
	inhibition	/	ECM	Suppression of metastasis	[124]

**Table 2:** Antioxidants reduce ROS levels in the body and inhibit tumors.

**Antioxidant Type**	**Tumor Type**	**Mechanism**	**References**
hyperoside	breast cancer	Hyperoside induces apoptosis in breast cancer cells	[155]
silibinin	breast cancer	Silibinin inhibits the migration and invasion of breast cancer cells	[156]
N-acetylcysteine	hepatocellular carcinoma	N-acetylcysteine reduces ROS stress defense against hepatocarcinogenesis	[157]
catalase	/	Catalase delivery inhibits tumor cell metastasis	[158]
multi-antioxidant (FTP)	breast cancer	Multi-antioxidant (FTP) scavenges ROS for metastatic breast cancer	[159]
allopurinol complex	breast cancer	Allopurinol complex reduces ROS levels to induce breast cancer cell death	[160]
vitamin E	liver cancer	Vitamin E reduces ROS production to inhibit the development of liver cancer	[161]
carotenoids	prostate, lung, and digestive cancers	Carotenoids inactivate ROS and protect against oxidative damage to prevent cancer	[162]
catalase	melanoma	Targeted delivery of catalase inhibits melanoma proliferation and metastasis	[163]

**Table 3:** Increasing ROS levels allows tumor cells to reach a critical state and induce inhibition or death.

**Oxidizer Type**	**Tumor Type**	**Mechanism**	**References**
astragalus	lung cancer	Astragalus enhances ROS production in cancer cells and inhibits cell migration and invasion of lung cancer cells	[164]
albanol B	lung cancer	Albanol B increased mitochondrial ROS production, inhibited proliferation, and induced apoptosis of lung cancer cells	[165]
lotus leaf flavonoids	lung cancer	Lotus leaf flavonoids increase ROS to induce apoptosis in lung cancer cells *in vitro*	[166]
chloroquine	cholangiocarcinoma	The autophagy inhibitor chloroquine increases intracellular ROS and increases the sensitivity of cisplatin in cholangiocarcinoma cells	[167]
fibulin 5 and beta1 integrins	pancreatic tumor	Loss of fibulin 5 binding to beta1 integrins inhibits tumor growth by increasing the level of ROS	[168]
oxaliplatin	oral squamous cell carcinoma	Oxaliplatin inhibits oral squamous cell carcinoma by increasing ROS production	[169]
chrysin	pancreatic cancer	Chrysin increases cellular ROS levels and induces ROS-dependent autophagy to induce pancreatic cancer cell death	[170]
flavonoids	colorectal cancer	Flavonoids increase the level of intracellular peroxide and induce apoptosis of colorectal cancer cells	[171]
ampelopsin	mammary tumor	Ampelopsin increases mitochondrial membrane permeability and induces mammary tumor cell apoptosis by increasing ROS levels	[172]
thymoquinone	/	Thymoquinone induces ROS production and prevents mediating cancer progression	[173]
gossypol	colorectal cancer	Gossypol induces ROS production and induces apoptosis of colorectal cancer cells	[174]
tempol	ovarian cancer	Tempol increase cellular ROS production and enhances cisplatin-induced apoptosis in ovarian cancer cells	[175]
melatonin	thyroid cancer	Melatonin induces reactive oxygen species to inhibit migration and induce apoptosis of thyroid cancer cells	[176]
ginsenoside Rg2	breast cancer	Ginsenoside Rg2 induces ROS production and enhances protein and mRNA expression of breast cancer apoptotic molecules	[177]
ascorbate	chronic lymphocytic leukemia	ROS accumulation by ascorbate enhances the cytotoxicity of ATO in chronic lymphocytic leukemia	[178]

**Table 4:** ROS action concentration.

**H_2_O_2_ (µM)**	**Microenvironmental Components**	**Direction of Action**	**References**
10	T cells	It interacts with T cells to promote the tumor microenvironment	[65]
50			[68]
10, 20, 50, 100			[69]
10, 50, 100			[66]
200, 400, 600, 800			[153]
1x10^4^, 5x10^4^			[152]
100		It interacts with T cells to suppress the tumor microenvironment	[75]
500			[74]
2	NK cells	It interacts with NK cells to suppress the tumor microenvironment	[83]
6.25-100		It interacts with NK cells to promote the tumor microenvironment	[82]
10			[65]
10, 20			[81]
100	macrophages	It interacts with macrophages to promote the tumor microenvironment	[84]
10, 10^-1^, 10^-3^	endothelial cells	It interacts with endothelial cells to promote the tumor microenvironment	[100]
3x10^3^			[97]
50	Fibroblast (associated with cancer)	It interacts with Fibroblast (associated with cancer) to suppress the tumor microenvironment	[115]
100	blood vessels	It interacts with blood vessels to suppress the tumor microenvironment	[121]
O^2•−^			
9-36μM NADH induction	Fibroblast	It interacts with Fibroblast (associated with cancer) to suppress the tumor microenvironment	[116]
500	blood vessels	It interacts with blood vessels to promote the tumor microenvironment	[119]
HO∙			
50, 100, 175, 250	blood vessels	It interacts with blood vessels to promote the tumor microenvironment	[120]
^1^O_2_			
2x10^-1^μM of MG2I induction	T cells	It interacts with T cells to promote the tumor microenvironment	[72]

## LIST OF ABBREVIATIONS

ROS: Reactive Oxygen Species
TME: Total Mesorectal Excision
